# Complete mitochondrial genome of *Callipogon relictus* Semenov (Coleoptera: Cerambycidae): a natural monument and endangered species in Korea

**DOI:** 10.1080/23802359.2017.1372718

**Published:** 2017-09-09

**Authors:** Jongok Lim, Dong-Keun Yi, Young Ho Kim, Wonhoon Lee, Sora Kim, Jung-Hoon Kang, Il-Kwon Kim

**Affiliations:** a Division of Forest Biodiversity, Korea National Arboretum, Pocheon, Korea;; b Korea Research Institute of Bioscience and Biotechnology, Daejeon, Korea;; c Department of Applied Biology, Kyungpook National University, Sangju, Korea;; d Department of Plant Medicine and Institute of Agriculture & Life Science, Gyeongsang National University, Jinju, Korea;; e Department of Agricultural Biotechnology, Laboratory of Insect Biosystematics, Seoul National University, Seoul, Korea;; f Research Institute of Agriculture and Life Sciences, Seoul National University, Seoul, Korea;; g Natural Heritage Center, National Research Institute of Cultural Heritage, Daejeon, Korea

**Keywords:** Korean natural monument, endangered species, mitogenome, longhorn beetle, *Callipogon relictus*

## Abstract

In the present study, we report the first complete mitochondrial DNA genome of the genus *Callipogon* based on *C. relictus*, a natural monument and endangered species in South Korea. The mitogenome is 15,742 base pairs with 13 protein coding genes (PCGs), two rRNAs, 22 tRNAs, and a 1033 bp long AT-rich region. The overall base composition was 67.3% AT and 32.7 GC. Among 13 PCGs, seven genes (*Nad2, Atp8, Atp6, Nad4L, Nad6, Cob, Nad1*) harbour the typical stop codon TAA or TAG, whereas remaining five genes terminate with T. Interestingly, *Cox3* employs AGA as the termination codon.

*Callipogon* is a genus with a disjunct distribution: one species, *C. relictus*, in Northeast Asia and seven in Central America (Cherepanov 1988; Lim, Kim, et al. 2013; Lim, Jung, et al. 2013). *Callipogon relictus* was designated as a Korean natural monument in 1968 and an endangered species in 2012 for a sharp decline of the population in Korea (Lim, Jung, et al. 2013).

A male and a female were found in Gwangneung forest, Korea in 2014 and 2015, respectively, and stored in absolute ETOH at −80 °C. The voucher specimens (accession no. NHC-A-2015-054, NHC-A-2016-120) are deposited in the Natural Heritage Center, Korea. We extracted genomic DNA from the bodies with DNA extraction kit (Takara, Japan). The extracted DNA sample was analysed using Illumina MiSeq. A total of 24,202,986 reads were analysed to generate 3,654,650,886 base pairs of sequence and assembled in Geneious 10.0.4 (Kearse et al. 2012). A total of 50,128 reads were assembled with an average coverage of 478.4Χ. Phylogenetic analysis was performed based on the nucleotide sequences of the 13 PCGs. Secondary structure prediction of tRNAs except for two tRNAs (tRNA*^Asn^* and tRNA*^Ser^*), was done in Lowe Lab tRNAscan-SE Search Server (Schattner et al. 2005) and the secondary structure prediction of tRNA*^Asn^* in Bioinformatics Web Server for RNA (http://rtools.cbrc.jp).

The *C. relictus* complete mitogenome, 15,742 bp, has 37 individual genes (13 protein coding genes (PCGs), two ribosomal RNAs, 22 tRNAs, and a 1033 bp long A + T rich region). The overall base composition was 67.3% AT and 32.7% GC. The 13 PCGs are similar to other coleopteran insects, and 12 genes begin with typical ATN codon: four (*Atp6, Cox3, Nad4L, Cob*) start with ATG, three (*Nad2, Nad3, Nad1*) with ATT, two (*Cox2, Nad6*) with ATC and three (*Atp8, Nad5, Nad4*) with ATA. ACC was observed as an atypical start codon for *Cox1*. Among 13 PCGs, seven genes (*Nad2, Atp8, Atp6, Nad4L, Nad6, Cob, Nad1*) harbour the typical stop codon TAA or TAG, whereas remaining five genes terminate with T. Interestingly, *Cox3* employs AGA as the termination codon. Taken together, one gene (*Cox1*) starts atypical codon, and 6 genes (*Cox1, Cox2, Cox3, Nad3, Nad5, Nad4*) possess atypical stop codon. The mitogenome of *C. relictus* has 22 tRNA genes: two isotypes for *Leu* and *Ser*, one type for the other 18 types of amino acid. With the exception of tRNA*^ser^*, the dihydrouridine arm of which forms a simple loop, 21 tRNAs can be folded into the typical clover-leaf structure. While the order of tRNA*^Ala^* and tRNA*^Arg^* among the ARNSEF (*Ala, Arg, Asn, Ser, Glu, Phe*) cluster between the *Nad3* and *Nad5* genes is reversed in some chrysomelid beetles and aquatic elateriform beetles (Timmermans and Vogler 2012; Timmermans et al. 2015). The lengths of 16S and 12S rRNA genes are 1287 and 817 bp, respectively, which are well within the range of the respective genes detected in the coleopteran mitochondrial rRNAs (Kim et al. 2009; Chiu et al. 2016). A large non-coding AT-rich region (79.6%) of 1033 nucleotides is located between 12S rRNA and tRNA*^Ile^*. Although certain repetitive sequences were not investigated in AT-rich region in *C. relictus*, a 16 bp-long poly-A, a 9 bp-long poly-T, and microsatellite-like TA repeats were scattered throughout this region (Table 1).

A phylogenetic position of *C. relictus* was inferred, and the analysis clearly showed the beetle as a distinct species among the other cerambycid species (Figure 1).

**Figure 1.**
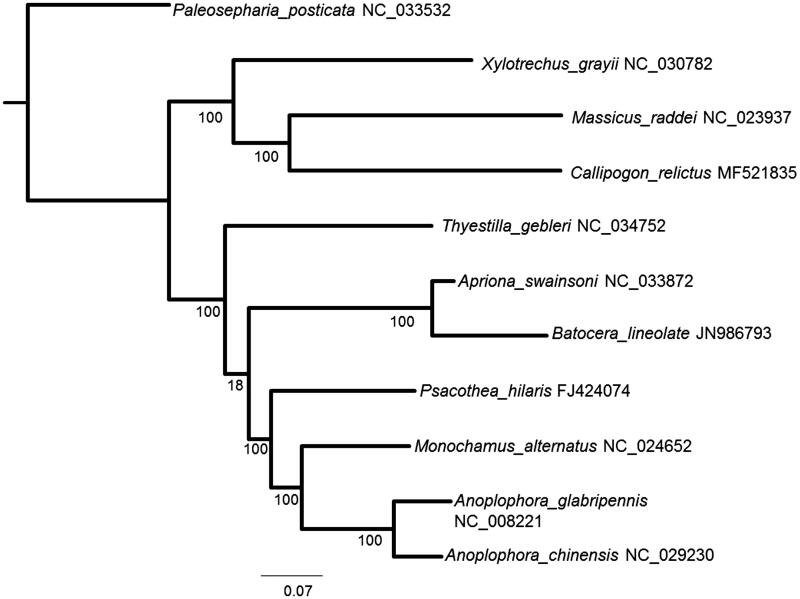
A maximum-likelihood tree inferred from the nucleotide sequences of 13 PCGs in the mitochondrial genome. The numbers beside the nodes are percentages of 1000 bootstrap values. *Paleosepharia posticata* was used as an outgroup. Alphanumeric terms indicate the GenBank accession numbers.

**Table 1.** Mitochondrial genome of *Callipogon relictus*.

Gene	Direction	Location	Length	Anticodon	Codon	
					Start	Stop
*tRNA^Ile^*	F	1–67	67	GAT (29–31)		
*tRNA^Gln^*	R	70–140	71	CAA (108–110)		
*tRNA^Met^*	F	143–211	69	CAT (173–175)		
*Nad2*	F	212–1225	1014		ATT	TAA
*tRNA^Trp^*	F	1224–1291	68	TCA (1258–1260)		
*tRNA^Cys^*	R	1292–1357	66	GCA (1326–1328)		
*tRNA^Tyr^*	R	1358–1422	65	GTA (1391–1393)		
*Cox1*	F	1415–2957	1513		ACC	T
*tRNA^Leu^*	F	2958–3022	65	TAA (2987–2989)		
*Cox2*	F	3023–3707	685		ATC	T
*tRNA^Lys^*	F	3708–3777	70	TTT (3737–3739)		
*tRNA^Asp^*	F	3778–3844	67	GTC (3809–3811)		
*Atp8*	F	3845–4000	156		ATA	TAA
*Atp6*	F	3994–4665	672		ATG	TAA
*Cox3*	F	4665–5453	786		ATG	AGA
*tRNA^Gly^*	F	5451–5515	65	TCC (5482–5484)		
*Nad3*	F	5516–5867	352		ATT	T
*tRNA^Ala^*	F	5868–5933	66	TGC (5897–5899)		
*tRNA^Arg^*	F	5930–5998	69	TCG (5962–5964)		
*tRNA^Asn^*	F	5999–6064	66	GTT (6031–6033)		
*tRNA^Ser^*	F	6064–6131	68	TCT (6096–6098)		
*tRNA^Glu^*	F	6132–6194	63	TTC (6162–6164)		
*tRNA^Phe^*	R	6193–6255	63	GAA (6223–6225)		
*Nad5*	R	6256–7978	1723		ATA	T
*tRNA^His^*	R	7976–8040	65	GTG (8007–8009)		
*Nad4*	R	8041–9370	1330		ATA	T
*Nad4L*	R	9367–9651	285		ATG	TAA
*tRNA^Thr^*	F	9654–9717	64	TGT (9684–9686)		
*tRNA^Pro^*	R	9718–9782	65	TGG (9750–9752)		
*Nad6*	F	9784–10,299	516		ATC	TAA
*Cob*	F	10,299–11,438	1140		ATG	TAG
*tRNA^Ser^*	F	11,437–11,503	67	TGA (11,466–11,468)		
*Nad1*	R	11,521–12,465	945		ATT	TAG
*tRNA^Leu^*	R	12,472–12,536	65	TAG (12,505–12,507)		
*16S rRNA*	R	12,538–13,824	1287			
*tRNA^Val^*	R	13,825–13,892	68	TAC (13,861–13,863)		
*12S rRNA*	R	13,893–14,709	817			
A + T rich region	R	14,710–15,742	1033			

Prior to the initiation of a conservation breeding program, the complete mitogenome of *C. relictus* from this study will be used to determine the population genetic structure and diversity among the species populations from all three countries.

## Nucleotide sequence accession number

The complete mitochondrial genome sequence of *Callipogon relictus* has been assigned GenBank accession number MF521835.
